# The EGR1/caspase-14/HIF-1α axis mediates tamoxifen resistance in MCF-7 breast cancer cells

**DOI:** 10.1515/biol-2025-1311

**Published:** 2026-05-11

**Authors:** Feihu Ji, Anping Zhao, Qiuxu Chen, Chenwei Li, Liyuan Huang

**Affiliations:** Department of Clinical Laboratory, The People’s Hospital of Yubei District of Chongqing, Chongqing, 401120, China; Department of Legal, The People’s Hospital of Yubei District of Chongqing, Chongqing, 401120, China; Department of Clinical Laboratory, Bishan Hospital of Chongqing Medical University, Bishan Hospital of Chongqing, Chongqing, 402760, China

**Keywords:** breast cancer, tamoxifen resistance, caspase14, EGR1/HIF-1α axis, HIF-1α

## Abstract

Breast cancer therapy is compromised by widespread tamoxifen resistance. The modulatory role of Caspase14 in the early growth response protein 1 (EGR1)/hypoxia-inducible factor 1-alpha (HIF-1α) axis in this context is unclear. We investigated how Caspase14 regulates EGR1/HIF-1α to promote 4-hydroxytamoxifen (4-OHT) resistance in MCF7 cells using cell-based genetic and biochemical assays (small-interference RNA knockdown, plasmid overexpression, Western blotting, qPCR, IC50 assays, and Chromatin immunoprecipitation experiments) across multiple independent experiments. Caspase14 expression correlated with resistance, with IC_50_ rising from 0.178 µM to 1.575 µM in resistant variants. EGR1 knockdown reduced Caspase14 and downstream targets GLUT3 and BCRP, implicating EGR1 as a key mediator. Resistant cells displayed elevated HIF-1α that enhanced metabolism and migration, further supporting a role in drug resistance. These data indicate that Caspase14 promotes survival, proliferation, and metabolic reprogramming in resistant MCF7 cells via the EGR1/HIF-1α pathway. Our findings reveal a mechanistic link between Caspase14 and endocrine therapy resistance and nominate Caspase14 as a therapeutic target to overcome tamoxifen resistance, with potential translational relevance.

## 1 Introduction

Breast cancer remains a prevalent malignancy affecting women globally. The recent estimation by the World Health Organization indicates that approximately 2.3 million new cases are diagnosed each year. This alarming trend underscores the urgency for effective therapeutic strategies. Moreover, in addition to being the most frequently diagnosed cancer, breast cancer is the leading cause of cancer-related mortality among women. The complexity of breast cancer is heightened by its heterogeneity, which encompasses distinct molecular subtypes, each with unique biological behavior and therapeutic responses. Consequently, the emergence of drug resistance significantly challenges the management of this disease, contributing to treatment failure and adversely affecting patient survival rates and quality of life [1], 2].

The understanding of breast cancer biology has evolved recently, revealing that hormone receptor-positive subtypes, particularly estrogen receptor-positive (ER+) breast cancers, usually develop resistance to endocrine therapies such as tamoxifen and aromatase inhibitors. This resistance arises through various mechanisms, including alterations in hormone signaling pathways, changes in tumor microenvironment, and the activation of compensatory survival pathways. Epidemiological studies indicate that approximately 30–50 % of patients with ER + breast cancer experience disease recurrence, primarily owing to acquired resistance to endocrine therapy [3], 4]. Such statistics highlight the urgency for the innovative elucidation of the underlying mechanisms of resistance, as well as the identification of novel therapeutic targets.

Early growth response protein 1 (EGR1) is an immediate-early gene product and a zinc-finger transcription factor that is rapidly induced by diverse stimuli, including growth factors, hypoxia, and cellular stress. It functions as a pivotal regulator of cell proliferation, differentiation, and apoptosis by modulating the expression of downstream target genes involved in these processes [5], 6]. In the context of cancer, EGR1 exhibits a complex, context-dependent role. It can act as a tumor suppressor by promoting apoptosis in some settings, yet substantial evidence highlights its pro-oncogenic functions in various malignancies, including breast cancer [7]. For instance, EGR1 has been shown to enhance breast cancer cell proliferation, survival, and metastasis through transcriptional activation of genes associated with these phenotypes [7], 8]. Importantly, emerging studies link EGR1 to the development of therapy resistance. It can transcriptionally upregulate multidrug resistance-associated proteins and integrin β1, thereby contributing to chemoresistance and metastatic potential in cancer cells [9], 10]. Furthermore, EGR1 is a known mediator of cellular responses to hypoxia and can interact with hypoxia-inducible pathways [11]. Given its established role in oncogenesis and stress adaptation, we hypothesized that EGR1 could serve as a critical upstream regulator within the signaling network driving tamoxifen resistance in breast cancer cells, potentially through its interaction with novel effectors such as Caspase14.

Current research efforts predominantly focus on the role of estrogen signaling in breast cancer pathogenesis, particularly its influence on tumor growth and survival. However, emerging evidence suggests that additional pathways may contribute to the development of resistance, including those involving key regulatory proteins such as Caspase14. Despite its critical role in apoptosis and inflammation, the involvement of Caspase14 in breast cancer, particularly in drug resistance, remains under explored. Moreover, the interaction of Caspase14 with transcription factors such as EGR1 and hypoxia-inducible factor 1-alpha (HIF-1α), which have been implicated in cellular responses to hypoxia and stress, presents a novel area of investigation that could provide insights into the mechanisms driving resistance in MCF7 breast cancer cells [12], 13]. The research landscape is marked by a notable gap concerning the specific regulatory roles of Caspase14 within the EGR1/HIF-1α signaling axis in MCF7 cells under the influence of 4-hydroxytamoxifen (4-OHT). While previous studies have established the significance of EGR1 and HIF-1α in various cancer types, their relationship with Caspase14 and the implications for drug resistance in MCF7 cells remain underexplored. Therefore, we aimed to elucidate the regulatory mechanisms through which Caspase14 modulates the EGR1 and HIF-1α pathways, ultimately influencing the response of MCF7 cells to 4-OHT treatment. This exploration holds potential for advancing the understanding of breast cancer biology may inform the identification of new therapeutic strategies aimed at overcoming drug resistance [13], 14].

To achieve our research objectives, we have employed a multifaceted methodological approach. This method included the use of cell culture techniques to maintain MCF7 cells, targeted gene silencing via small-interference (si)RNA to assess the role of Caspase14, and plasmid constructs for overexpression studies. Additionally, we used Western blotting and quantitative PCR to evaluate the expression levels of key proteins and mRNA associated with EGR1 and HIF-1α pathways, respectively. Furthermore, we conducted IC_50_ assays to determine the sensitivity of MCF7 cells to 4-OHT in the context of altered Caspase14 expression. Moreover, chromatin immunoprecipitation (ChIP) assays were used to investigate the binding interactions between Caspase14 and the promoters of EGR1 and HIF-1α, providing insights into the transcriptional regulation mechanisms [15].

This study is poised to contribute significantly to the field of breast cancer research by elucidating the role of Caspase14 in modulating EGR1 and HIF-1α signaling pathways and their collective impact on drug resistance in MCF7 cells. By identifying these interactions, we aim to inform the development of targeted therapeutic strategies that could improve patient outcomes in breast cancer treatment. Our findings may offer valuable insights that extend beyond the realm of breast cancer, informing broader oncological research and therapeutic approaches. This study addresses a critical gap in current knowledge and holds promise for advancing clinical practices in the management of breast cancer.

## 2 Methods

### 2.1 Cell culture and reagents

MCF7, a breast cancer cell was obtained from the ATCC (Manassas, VA) and stored in liquid nitrogen. The MCF7 was cultured in DMEM containing 10 % FBS, penicillin (50 U/ml) and streptomycin (100 μg/ml) at 37 °C in a 5 % CO_2_ atmosphere. 4-OHT was obtained from Sigma-Aldrich (St. Louis, MO, USA). The antibody against EGR1 (4153), estrogen receptor alpha (ERα) (8644), Bcl-2 (15071) and Bax (5023) were obtained from Cell Signaling, Inc. (Beverly, MA, USA). The antibody against caspase 14 and glucose transporter 3 (GLUT3) were obtained from GeneTex (San Antonio, TX, USA). The antibody against breast cancer resistance protein (BCRP) (ab3380) was purchased from Abcam (Waltham, MA, USA). The antibody against HIF-1α(NB100-105) was obtained from Novus Biologicals (Centennial, CO, USA).

### 2.2 Establishment of 4-OHT resistant MCF7 cell line

To establish a 4-OHT resistant cell line (TAM-R), cells were allowed to adhere and reach approximately 70–80 % confluence. Subsequently, 0.1 µM 4-OHT was added to the culture medium. The medium was replaced every 48–72 h, and cells were monitored for growth and morphological changes. After the cells adapted and their growth normalized, the 4-OHT concentration was gradually increased every 2 weeks: initially to 0.2 µM, subsequently to 0.5 µM, and finally to 1 µM, maintaining each concentration until the cell growth stabilized. After maintaining 1 µM 4-OHT for 6 months, the cells were continuously cultured in this medium to sustain resistance. Throughout this process, cells were sub-cultured at 80–90 % confluence, and the morphology and growth rates were monitored regularly.

### 2.3 Western blotting

After collecting the cells, whole-cell lysates were prepared in RIPA buffer supplemented with protease/phosphatase inhibitors and quantified by BCA assay. Equal amounts of protein were resolved by SDS-PAGE and transferred to PVDF membranes. After blocking with 5 % nonfat milk or BSA in TBST, membranes were incubated with primary antibodies against EGR1, CASP14, ERα, HIF-1α, GLUT3, BCRP (ABCG2), BCL2, and BAX at 4 °C overnight, followed by HRP-conjugated secondary antibodies. Bands were visualized by enhanced chemiluminescence and imaged. Densitometry was performed with normalization to GAPDH, and data represent at least three independent experiments.

### 2.4 Quantitative real-time PCR

In this study, we performed RT-qPCR experiments using MCF7 cells to investigate the expression levels of genes including caspase14, ESR1, ABCG2, bcl2, and bax. Total RNA was extracted from cultured MCF7 cells using TRIzol Reagent according to the manufacturer’s instructions for cell lysis and RNA purification. Following RNA extraction, the concentration and purity of the RNA were assessed using a NanoDrop spectrophotometer. To eliminate potential genomic DNA contamination, DNase I treatment was applied when necessary. Subsequently, 1 μg of RNA was reverse transcribed into cDNA using a reverse transcription kit, following the manufacturer’s protocol. The qPCR reaction mixture comprised SYBR Green qPCR Master Mix, specific primers for each target gene, and the cDNA template. Amplification was carried out using a standard thermal cycling program, and the specificity of the amplification was confirmed via melt curve analysis. Data normalization was performed using housekeeping genes such as GAPDH, and the relative expression levels of the target genes were calculated using the 2^−ΔΔCt^ method. All experiments were conducted in triplicate to ensure reproducibility. The primers used for RT-qPCR were list in Table 1.

**Table 1:** Human gene primer in this research were listed below.

Gene		Primer sequence (5′–3′)
CASP14	F	CTG​GTG​GAT​GTG​TTC​ACG​AAG​AGG
	R	AGG​GTG​CTT​TGG​ATT​TCA​GGG​TTC
ESR1	F	GTG​GGA​ATG​ATG​AAA​GGT​GGG​AT
	R	GGT​TGG​CAG​CTC​TCA​TGT​CTC
SLC2A3	F	TAT​GGC​CGC​TGC​TAC​TGG​GTT
	R	CAA​CCG​CTG​GAG​GAT​CTG​CTT
ABCG2	F	GAC​TTA​TGT​TCC​ACG​GGC​CT
	R	GGC​TCT​ATG​ATC​TCT​GTG​GCT​TT
bcl-2	F	AGG​ATA​ACG​GAG​GCT​GGG​ATG
	R	TGT​TGA​CTT​CAC​TTG​TGG​CCC
Bax	F	CCC​TTT​TGC​TTC​AGG​GTT​TCA​T
	R	AGA​CAC​TCG​CTC​AGC​TTC​TTG
GAPDH	F	GAC​ATG​CCG​CCT​GGA​GAA​AC
	R	AGC​CCA​GGA​TGC​CCT​TTA​GT

### 2.5 Chromatin immunoprecipitation (ChIP) assay

Cells were crosslinked with 1 % formaldehyde, quenched with glycine, lysed, and chromatin was sonicated to ∼200–500 bp. Equal amounts of sheared chromatin from each group were immunoprecipitated with an anti-EGR1 antibody or species-matched IgG control using protein A/G magnetic beads. After washing and elution, crosslinks were reversed and DNA was purified. qPCR was performed with SYBR Green using primers spanning predicted EGR1-binding sites within the human CASP14 promoter. The primers used for RT-qPCR were: 5′-GAA​CTT​GGC​CTT​GAG​GAA​CAG-3’ (forward) and 5′-TGC​CAA​AAC​TGG​CCA​GAG​C-3’ (reverse) for CASP14. Enrichment was quantified as percent input and as fold over IgG, and compared between the two cell groups. Assays were conducted in technical triplicates and in at least three independent biological replicates; where indicated, a known EGR1 target and a distal nonbinding region were included as positive and negative controls, respectively.

### 2.6 siRNA knockdown

MCF-7 parental cells and a TAM-R subline were seeded to ∼40–60 % confluence in antibiotic-free medium and transfected with CASP14-targeting siRNA (25–50 nM) using a lipid-based reagent (Lipofectamine RNAiMAX) following the manufacturer’s protocol. A non-targeting siRNA served as the negative control; a mock (reagent-only) control was included where indicated. Medium was replaced with complete growth medium after 6 h, and cells were harvested 24–72 h post-transfection. Knockdown efficiency was verified by qRT–PCR at 24 h and by immunoblotting at 48 h (normalized to GAPDH/ACTB). Expression of selected downstream genes were measured in parallel at the mRNA and/or protein level and analyzed relative to the non-targeting control (qPCR by the 2^−ΔΔCt^ method). All conditions were performed in at least three independent biological replicates, with qPCR in technical triplicates.

### 2.7 Plasmid transfection

TAM-R cells were seeded to approximately ∼50–70 % confluence in an antibiotic-free medium and transfected with an endotoxin-free HIF1A expression plasmid (CMV-driven) using the Lipofectamine 3000 obtained from Thermo Fisher Scientific (Waltham, MA, USA) according to the manufacturer’s instructions. An empty vector was used as the negative control; a mock (reagent-only) control was included where indicated. After 4–6 h, transfection mixtures were replaced with a complete medium, and cells were collected 24–72 h post-transfection. Overexpression was confirmed via RT–qPCR (24 h) and immunoblotting (48 h) with GAPDH as internal controls. Selected downstream targets were examined in simultaneously the mRNA and/or protein level and analyzed relative to the empty vector control (qPCR using the 2^−ΔΔCt^ method). Experiments were performed in at least three independent biological replicates.

### 2.8 Glucose consumption and lactate production

Cells were seeded at 5 × 10^4^ cells/well in 24-well plates in 1.0 mL DMEM. After a 24 h incubation, conditioned media were collected and glucose concentrations were measured using the Glucose Assay Kit-WST (Dojindo, Kumamoto, Japan) according to the manufacturer’s instructions. Cell numbers at the end of incubation were determined with a hematology analyzer. Glucose consumption was calculated as the decrease in glucose relative to fresh medium and normalized to the terminal cell count. Lactate production in culture supernatants was quantified using a Lactate Assay Kit (Nanjing Jiancheng Bioengineering Institute, Nanjing, China) according to the manufacturer’s instructions. Production was calculated as the increase over baseline, i.e., lactate concentration in conditioned medium collected at the specified time minus the lactate concentration in fresh (unused) medium.

## 3 Results

### 3.1 Differential sensitivity to 4-OHT and gene expression in MCF7 parental and resistant cells

**Figure 1:**
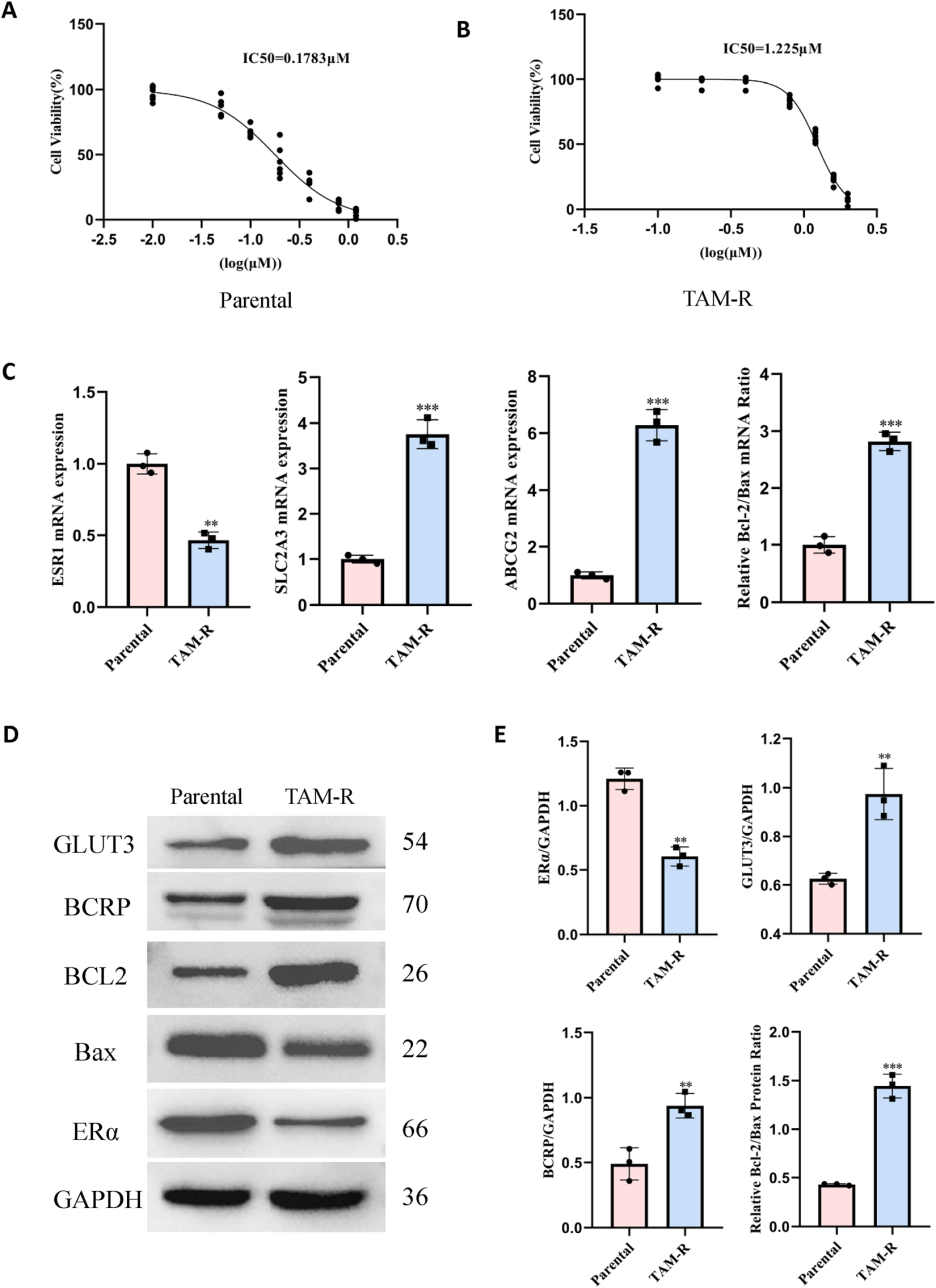
Characterization of tamoxifen resistance and associated molecular alterations in tamoxifen-resistant (TAM-R) cells. (A and B) Cell viability curves of MCF7 parental and TAM-R cells following treatment with increasing concentrations of 4-hydroxytamoxifen (4-OHT) for 72 h. (C) mRNA expression levels of the indicated genes as determined by RT-qPCR. Data were normalized to GAPDH and are presented as fold change relative to parental cells. (D) Representative western blotting images showing the protein expression levels of estrogen receptor alpha (ERα), glucose transporter 3 (GLUT3), breast cancer resistance protein (BCRP), BCL-2, and Bax. GAPDH was used as a loading control. (E) Densitometric quantification of the protein levels from western blotting analysis. All data are presented as mean ± SD of at least three separate experiments. P-values were calculated using a two-tailed Student’s *t*-test. **P < 0.01; ***P < 0.001 versus parental cells.

MCF7 parental cells were treated with varying concentrations of 4-OHT (0, 0.01, 0.05, 0.1, 0.2, 0.4, 0.8, and 1.2 µM) and MCF7 resistant (TAM-R) cells were treated with 0, 0.1, 0.2, 0.4, 0.8, 1.2, 1.6, and 2.0 µM 4-OHT. After 72 h of incubation, cell viability was assessed using the CCK8 assay to determine the IC_50_ values. The IC50 of MCF7 parental cells was 0.178 µM, while that of TAM-R cells was higher at 1.575 µM (Figure 1A and B).

Western blotting and RT-qPCR analyses revealed the expression levels of specific genes and proteins in the MCF7 parental and TAM-R cells. The genes and proteins analyzed included ERα, GLUT3, BCRP, BCL2, and Bax. Compared with those in the MCF7 parental cells, the TAM-R cells exhibited significantly elevated levels of GLUT3, and BCRP at the gene and protein levels; however, ERα was downregulated (Figure 1C–E). Additionally, the BCL2/Bax ratio at the gene and protein levels was significantly higher in the TAM-R cells, indicating a shift towards an anti-apoptotic profile.

### 3.2 Impact of Caspase14 knockdown on gene and protein expression in MCF7 parental and TAM-R cells

**Figure 2:**
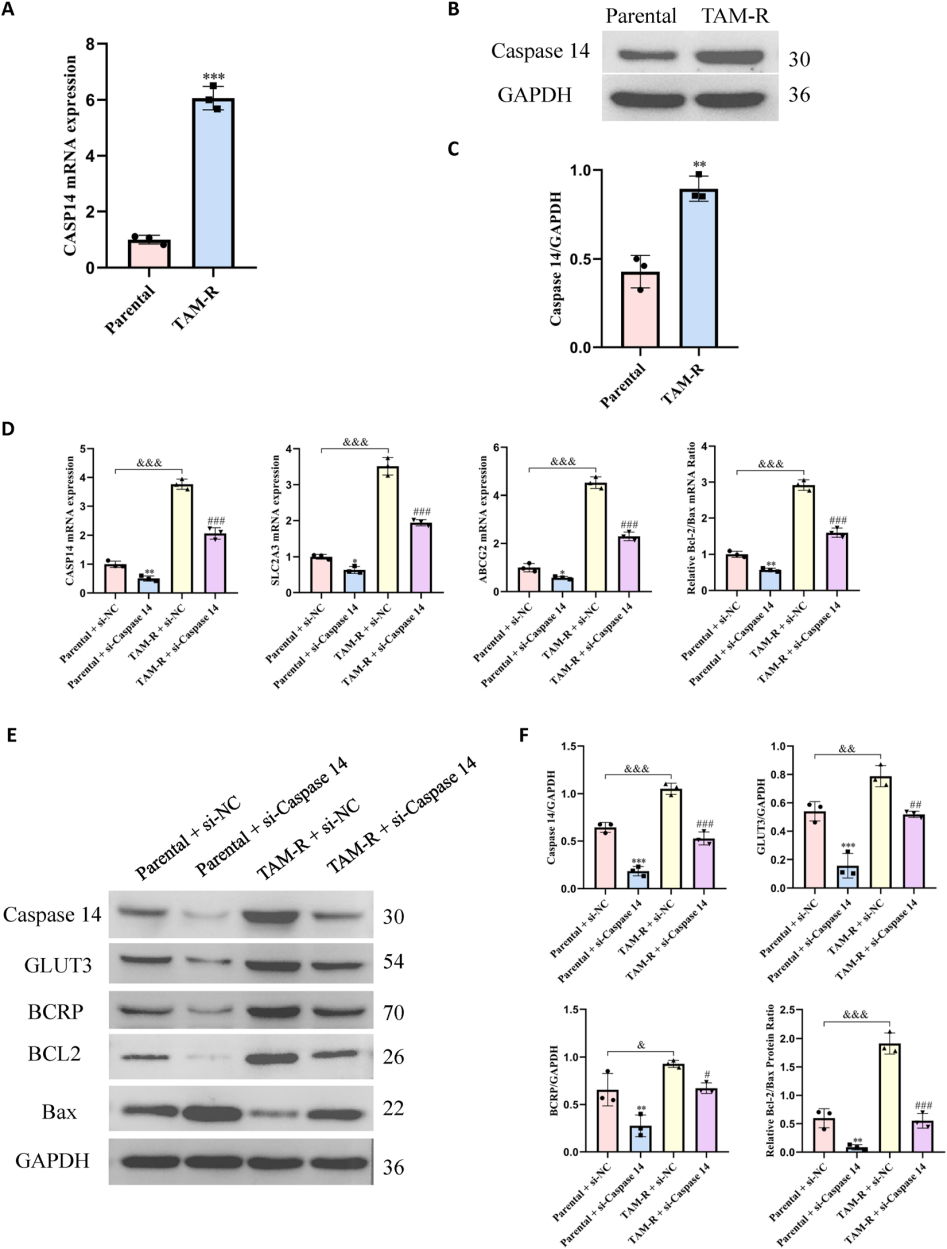
Caspase14 was upregulated in TAM-R cells, and its knockdown modulated the expression of resistance-associated markers. (A) mRNA expression level of Caspase14 as determined by RT-qPCR. (B and C) Representative western blotting images and densitometric analyses showing the protein expression levels of Caspase14 in MCF7 parental and TAM-R cells. Data were normalized to GAPDH and presented as fold change relative to parental cells. Data were presented as mean ± SD (*n* = 3); ***p < 0.001 versus parental cells (Student’s *t*-test). (D) mRNA expression levels of the indicated genes as determined by RT-qPCR. (E and F) Representative western blotting images and densitometric analyses showing the protein expression levels of caspase14, GLUT3, BCRP, Bcl-2, and Bax after *CASP14*-specific siRNA transfection. Data are presented as mean ± SD (*n* = 3); *P < 0.05; **P < 0.01; ***P < 0.001 versus the parental + control siRNA (si-NC) group; ^##^P < 0.01; ^###^P < 0.001 versus the TAM-R + si-NC group; ^&&^P < 0.01; ^&&&^P < 0.001 versus the parental + si-NC group (two-way ANOVA with post-hoc test for multiple comparisons).

Furthermore, western blotting and RT-qPCR revealed the expression levels of Caspase14 at the gene and protein levels in the MCF7 parental and TAM-R cells. Caspase14 gene and protein levels were significantly elevated in the TAM-R cells compared with those in the parental cells (Figure 2A–C).

To further explore the role of Caspase14, we used siRNA to knock down its expression in MCF7 parental and TAM-R cells. Following the knockdown, we assessed the expression levels of Caspase14, GLUT3, BCRP, BCL2, and Bax using western blotting and RT-qPCR. Caspase14 knockdown resulted in a significant reduction in the gene and protein expression levels of Caspase14, GLUT3, and BCRP in both cell lines (Figure 2D–F). In addition, the BCL2/Bax ratio at the gene and protein levels was significantly reduced following Caspase14 knockdown.

### 3.3 Enhanced migration, glucose uptake, and lactate production in Caspase14-Expressing TAM-R cells

**Figure 3:**
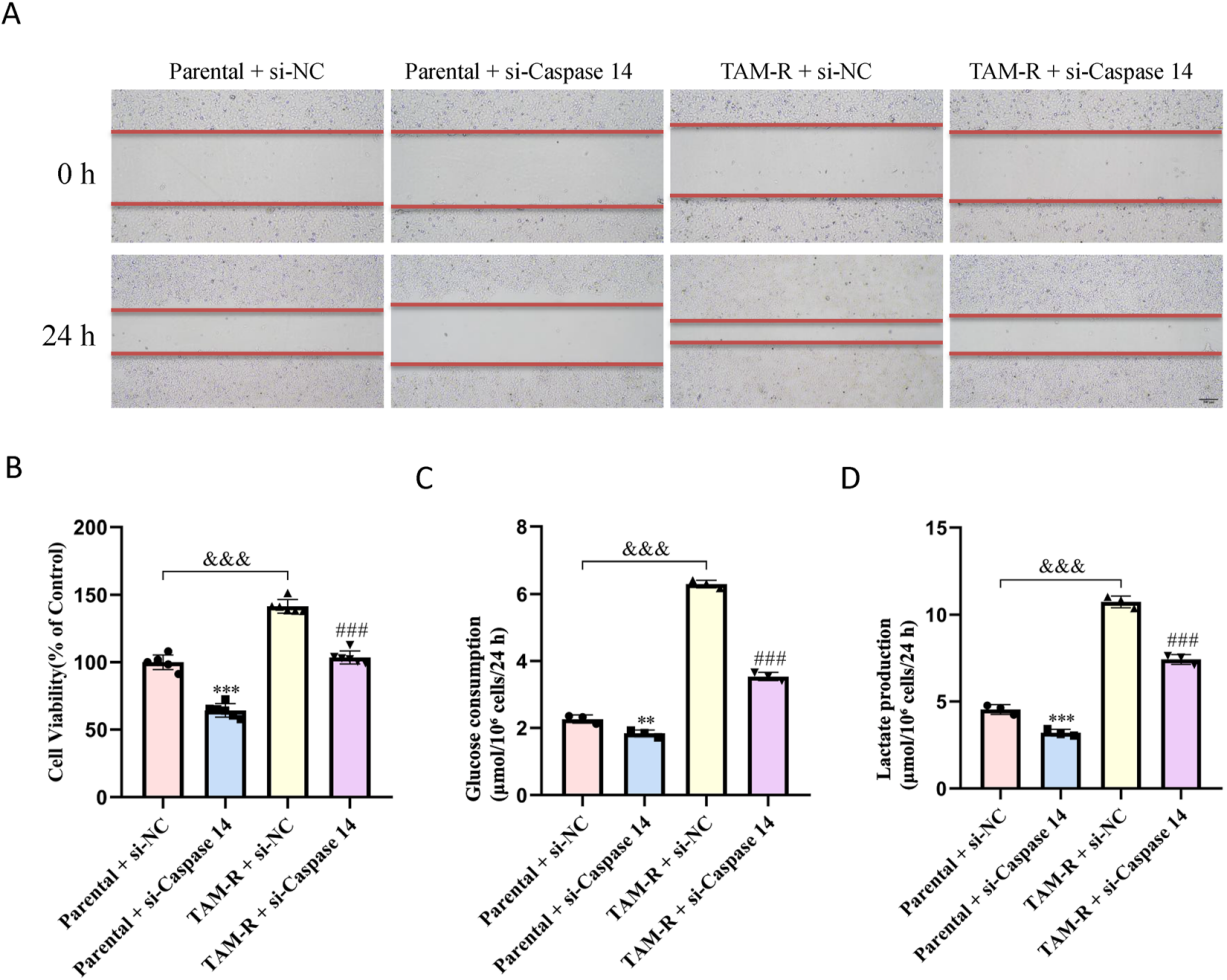
Caspase14 knockdown impairs migration, viability, and metabolic reprogramming in TAM-R cells. (A) Representative images of wound healing assays performed in MCF7 parental and TAM-R cells transfected with control si-NC or CASP14-specific siRNA (si-Caspase14). Scale bar = 200 μm. Data are representative of at least three independent experiments. (B) Cell viability was assessed using CCK-8 assay in the indicated cell lines and treatment groups after 72 h. (C) Glucose uptake was measured in MCF7 parental and TAM-R cells following siRNA-mediated knockdown of Caspase14. (D) Lactate production in the cell culture supernatant was quantified using a lactate assay kit. All data are presented as mean ± SD of at least three separate experiments. **P < 0.01; ***P < 0.001 versus the parental + si-NC group; ^###^P < 0.001 versus the TAM-R + si-NC group; ^&&&^P < 0.001 versus the parental + si-NC group (two-way ANOVA with post-hoc test for multiple comparisons).

The scratch wound healing assay and CCK8 assay revealed that the viability and migratory capacity and cell viability of TAM-R cells were significantly higher compared to MCF7 parental cells. Furthermore, Caspase14 knockdown via siRNA significantly reduced the viability and migratory ability of MCF7 parental and TAM-R cells (Figure 3A and B).

Additionally, glucose uptake and lactate production assays revealed the metabolic activity states. The TAM-R cells exhibited significantly higher glucose uptake and lactate production than did the parental cells. However, Caspase14 knockdown caused a marked reduction in glucose uptake and LDH release in both cell lines (Figure 3C and D). These findings suggest that Caspase14 plays a critical role in enhancing the drug resistance of TAM-R cells by promoting increased cell migration, glucose uptake, and lactate production.

### 3.4 Elevated EGR1 expression in TAM-R cells and its regulatory role on key genes and proteins

**Figure 4:**
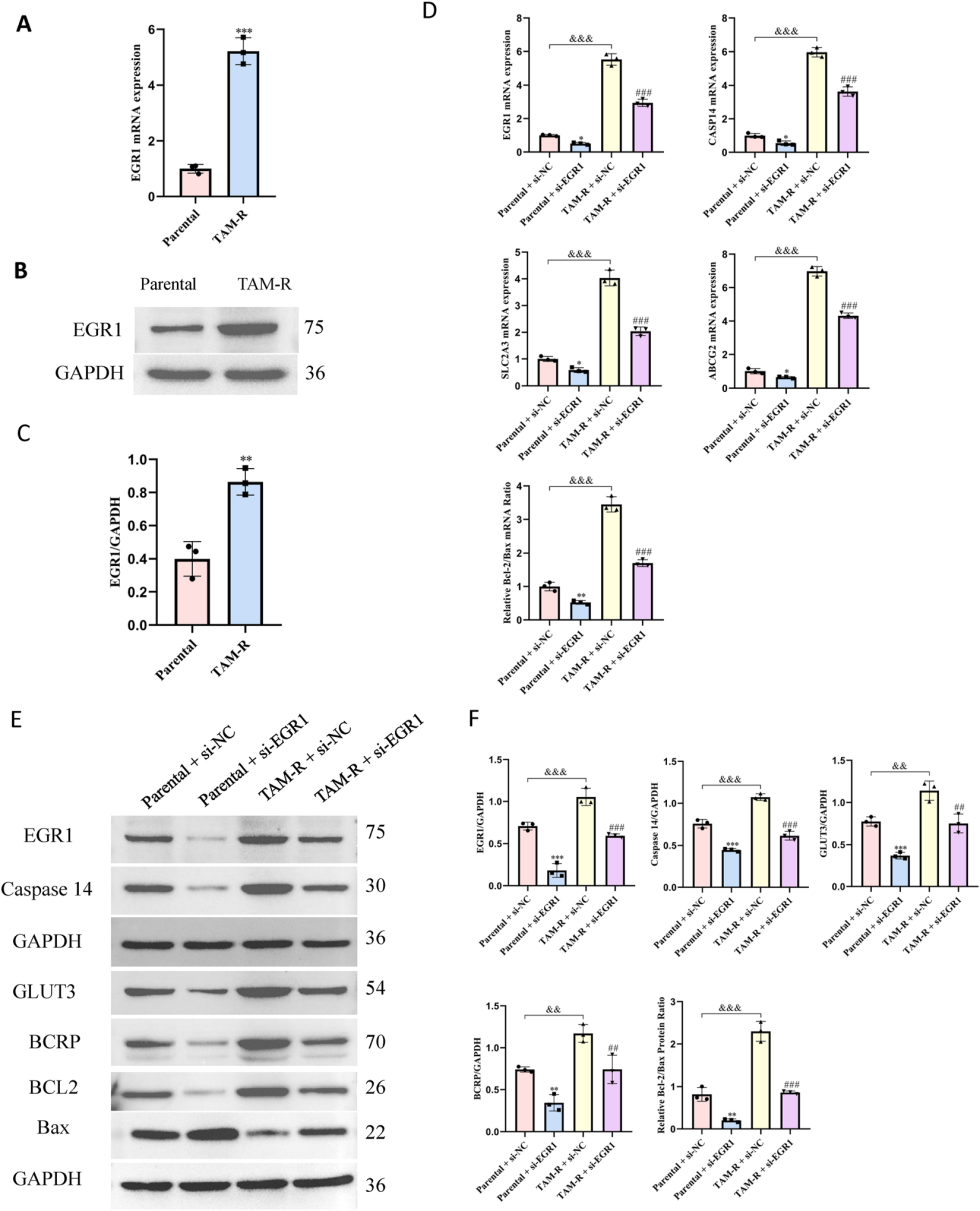
EGR1 regulates the expression of resistance-associated genes. (A) mRNA expression levels of Caspase14 as determined by RT-qPCR. (B and C) Representative western blotting images and densitometric analyses showing the protein levels of caspase14 in MCF7 parental and TAM-R cells. Data were normalized to GAPDH and presented as fold change relative to parental cells. Data are presented as the mean ± SD (*n* = 3); **p < 0.01; ***p < 0.001 versus parental cells (Student’s *t*-test). (D) mRNA expression levels of the indicated genes as determined by RT-qPCR. (E and F) Representative western blotting images and densitometric analyses showing the protein levels of EGR1, Caspase14, GLUT3, BCRP, BCL-2, and Bax after *CASP14*-specific siRNA transfection. Data are presented as mean ± SD (*n* = 3); *P < 0.05; **P < 0.01; ***P < 0.001 versus the parental + si-NC group; ^##^P < 0.01; ^###^P < 0.001 versus the TAM-R + si-NC group; ^&&^P < 0.01; ^&&&^P < 0.001 versus the parental + si-NC group (two-way ANOVA with post-hoc test for multiple comparisons).

Western blotting and RT-qPCR showed that EGR1 expression at the gene and protein levels was significantly higher in the TAM-R cells than in the MCF7 parental cells (Figure 4A–C).

siRNA-mediated EGR1 knockdown in MCF7 parental and TAM-R cells revealed the role of EGR1. The post-knockdown expression levels of EGR1, Caspase14, GLUT3, BCRP, BCL2, and BAX were revealed by western blotting and RT-qPCR. EGR1 knockdown led to a significant reduction in the gene and protein expression levels of Caspase14, ERα, GLUT3, and BCRP in both cell lines (Figure 4D–F). Additionally, the BCL2/Bax ratio at the gene and protein levels was significantly reduced following EGR1 knockdown. These findings suggest that EGR1 plays a crucial role in regulating the expression of key genes and proteins associated with drug resistance in TAM-R cells.

### 3.5 EGR1 knockdown attenuated cell migration and resistance by transactivating Caspase14 expression

**Figure 5:**
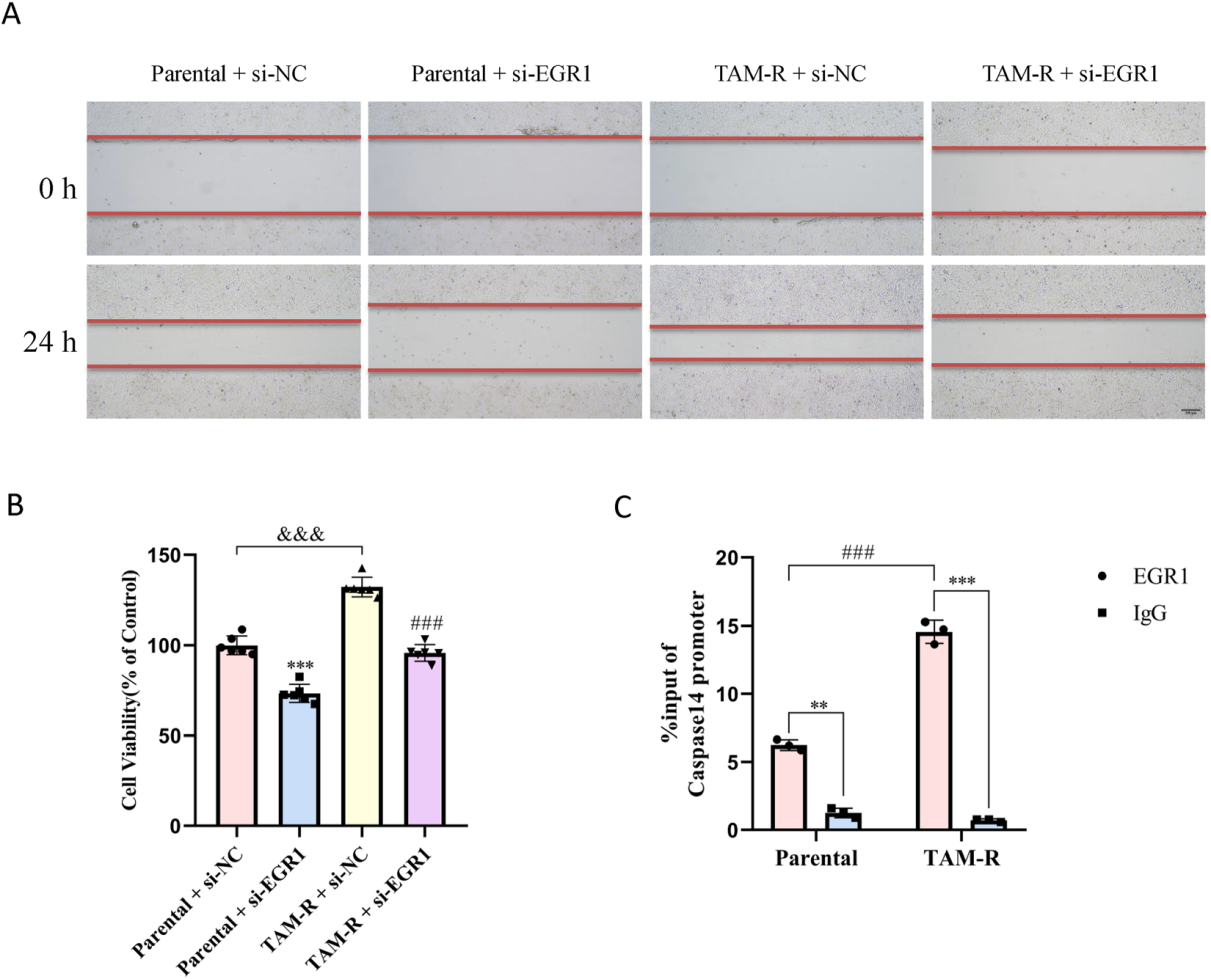
EGR1 drives migration and resistance by directly binding to the Caspase14 promoter. (A) Representative images of wound healing assays performed on MCF7 parental and TAM-R cells transfected with si-NC or EGR1-specific siRNA (si-EGR1). Scale bar = 200 μm. Data are representative of at least three independent experiments. (B) Cell viability was assessed by CCK-8 assay in the indicated cell lines and treatment groups after 72 h. Data are presented as mean ± SD (*n* = 6); ***P < 0.001 versus the parental + si-NC group; ^###^P < 0.001 versus the TAM-R + si-NC group group; ^&&&^P < 0.001 versus the parental + si-NC group (two-way ANOVA with post-hoc test for multiple comparisons). (C) Chromatin immunoprecipitation (ChIP) assays were performed in MCF7 parental and TAM-R cells using an anti-EGR1 antibody or normal IgG (negative control). The precipitated DNA was analyzed by qPCR with primers specific for the putative EGR1-binding site(s) in the human *Caspase14* promoter region. Data are presented as fold enrichment relative to the IgG control ± SD (*n* = 3). **p < 0.01, ***p < 0.001 versus anti-EGR1 antibody group; ^###^P < 0.001 versus parental group (two-way ANOVA).

Using siRNA to knock down EGR1, we observed a significant reduction in the migratory capacity and viability of the MCF7 parental and TAM-R cells (Figure 5A and B). This finding underscores the potential role of EGR1 in cell migration and cell viability and its contribution to cancer progression.

The role of EGR1 in cancer progression has been extensively documented, including its involvement in gastric and colorectal cancers [16]. We further elucidated the mechanism by which EGR1 regulates tamoxifen resistance through Caspase14. The ChIP assays revealed the binding relationship between EGR1 and the caspase14 promoter. EGR1 bound to the Caspase14 promoter region in the MCF7 parental and TAM-R cells, with significantly higher binding observed in the TAM-R cells than in the parental cells (Figure 5C).

These findings suggest that EGR1 enhances the migratory capacity of TAM-R cells and potentially contributes to tamoxifen resistance by interacting with the Caspase14 promoter, highlighting a novel regulatory pathway in drug-resistant breast cancer cells.

### 3.6 HIF-1α knockdown reduces expression of key genes and proteins involved in tamoxifen resistance in MCF7 cells

**Figure 6:**
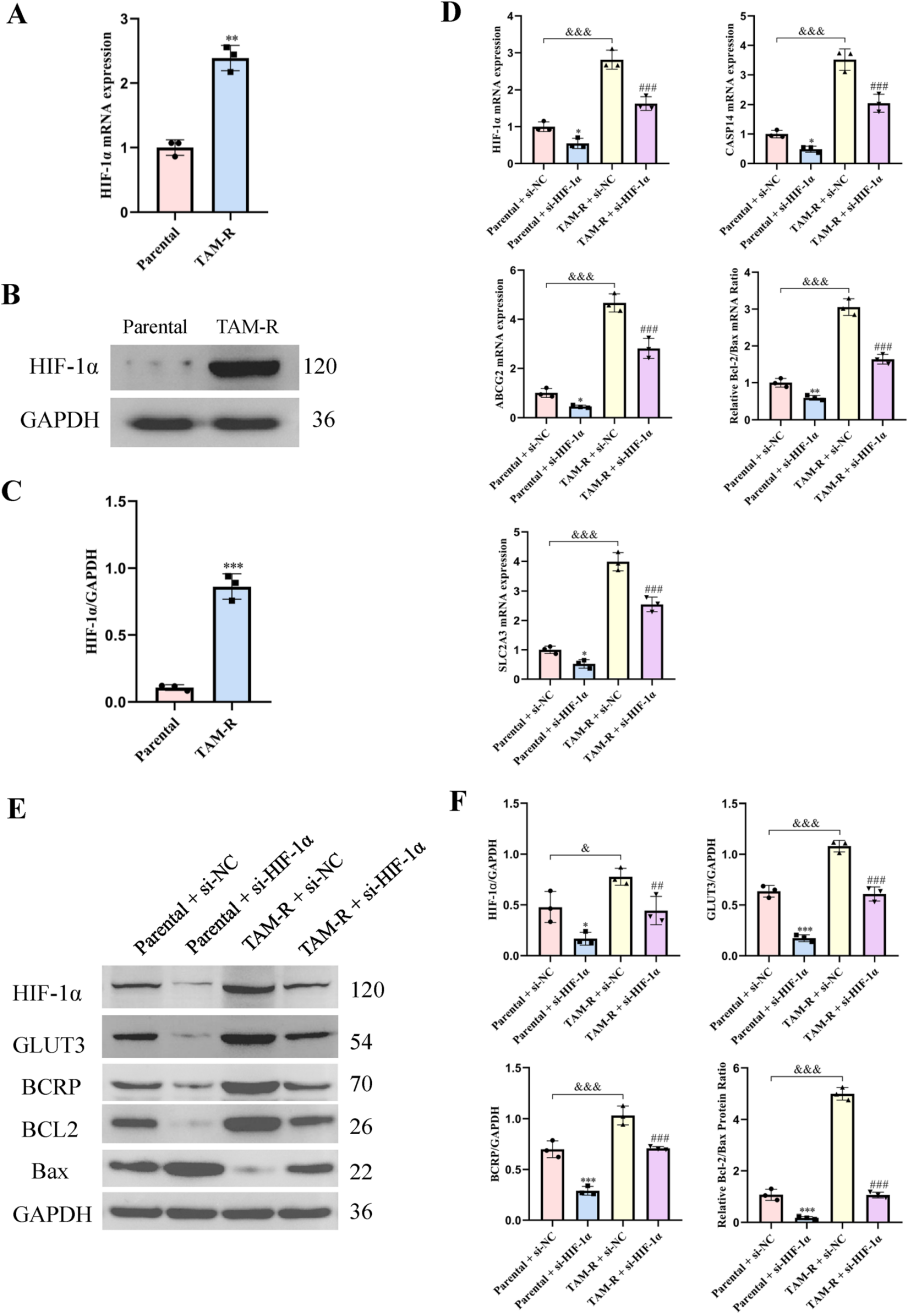
HIF-1α modulates tamoxifen resistance-related genes in MCF7 cells. (A) mRNA expression level of HIF-1α as determined by RT-qPCR. (B and C) Representative western blotting images and densitometric analyses showing the protein levels of HIF-1α in MCF7 parental and TAM-R cells. Data were normalized to GAPDH and presented as fold change relative to parental cells. Data are presented as mean ± SD (*n* = 3); **p < 0.01; ***p < 0.001 versus parental cells (Student’s *t*-test). (D) mRNA expression levels of the indicated genes, as determined via RT-qPCR. (E and F) Representative western blotting images and densitometric analyses showing the protein levels of HIF-1α, GLUT3, BCRP, Bcl-2, and Bax after *HIF-1*-specific siRNA transfection. Data are presented as mean ± SD (*n* = 3); *P < 0.05; **P < 0.01; ***P < 0.001 versus the parental + si-NC group; ^###^P < 0.001 versus the TAM-R + si-NC group; ^&^P < 0.05; ^&&&^P < 0.001 versus the parental + si-NC group (two-way ANOVA with post-hoc test for multiple comparisons).

HIF-1α has been implicated in the development of drug resistance across various cancers [17]. We explored the role of HIF-1α in mediating tamoxifen resistance in MCF7 cells. Western blotting and RT-qPCR revealed that the expression of HIF-1α at the gene and protein levels was higher in the TAM-R cell lines than in the MCF7 parental cells (Figure 6A–C).

siRNA-mediated knockdown of HIF-1α in the MCF7 parental and TAM-R cells revealed the HIF-1α role. Western blotting and RT-qPCR showed the changes in the expression levels of GLUT3, BCRP, BCL2, and Bax after knocking down HIF-1α. HIF-1α knockdown caused a significant reduction in the gene and protein expression levels of GLUT3 and BCRP in both cell lines (Figure 6D–F). Additionally, the BCL2/Bax ratio at the gene and protein levels was notably decreased following HIF-1α knockdown.

These findings suggest that HIF-1α plays a crucial role in regulating the expression of key genes and proteins associated with tamoxifen resistance in MCF7 cells, highlighting its potential as a therapeutic target for overcoming drug resistance in breast cancer.

### 3.7 Reduced HIF-1α expression suppresses tamoxifen resistance in MCF7

**Figure 7:**
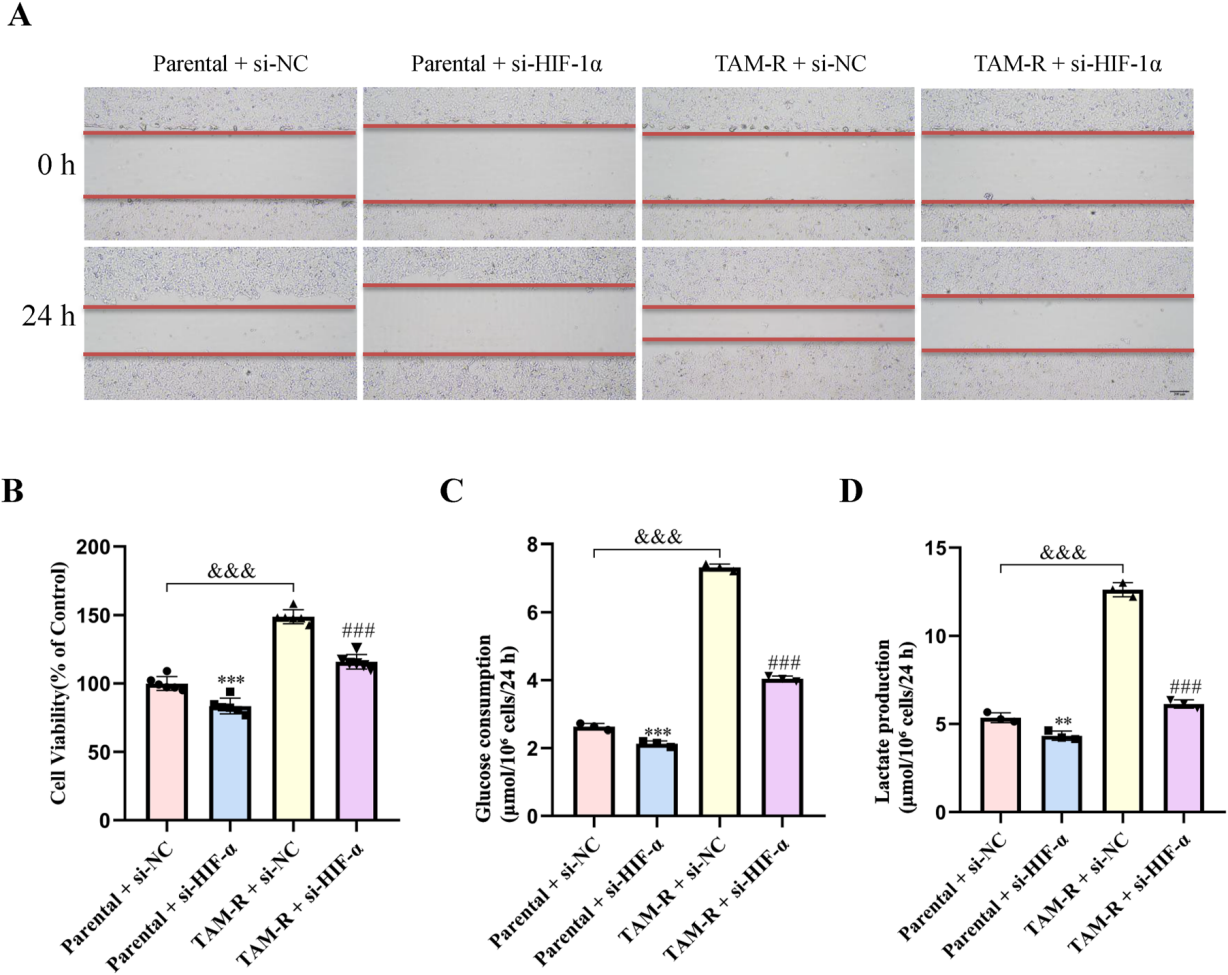
HIF-1α knockdown suppressed migration, viability, and glycolytic metabolism in MCF7 cells. (A) Representative images of wound healing assays performed in MCF7 parental and TAM-R cells transfected with si-NC or *HIF-1α*-specific siRNA (si-HIF-1α). Scale bar = 200 μm. Data are representative of at least three independent experiments. (B) Cell viability was assessed using the CCK-8 assay in the indicated cell lines and treatment groups after 72 h. (C) Glucose uptake was measured in MCF7 parental and TAM-R cells following siRNA-mediated knockdown of HIF-1α. (D) Lactate production in the cell culture supernatant was quantified using a lactate assay kit. All data are presented as mean ± SD of at least three separate experiments. **P < 0.01; ***P < 0.001 versus the parental + si-NC group; ^###^P < 0.001 versus the TAM-R + si-NC group; ^&&&^P < 0.001 versus the parental + si-NC group (two-way ANOVA with post-hoc test for multiple comparisons).

siRNA-mediated knockdown of HIF-1α caused a significant reduction in the viability and migratory capacity of the MCF7 parental and TAM-R cells (Figure 7A and B). Additionally, glucose uptake and lactate release were notably decreased in both cell lines following HIF-1α knockdown (Figure 7C and D). These findings suggest that HIF-1α significantly influences cell migration, cell viability, glucose metabolism, and tamoxifen resistance in MCF7 cells.

### 3.8 HIF-1α participated in the EGR1/Caspase14 pathway-regulated tamoxifen resistance in the MCF7 cells

**Figure 8:**
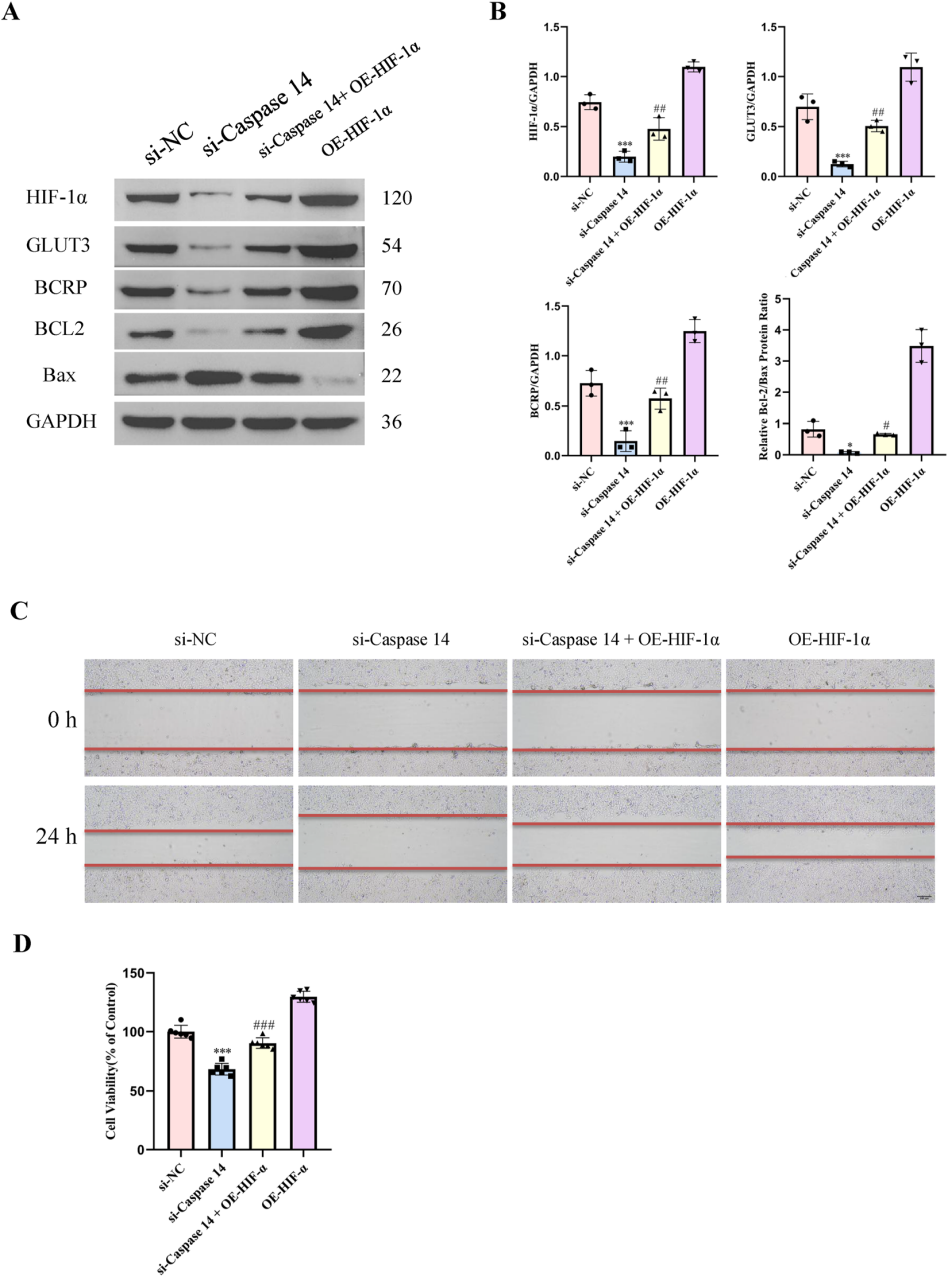
HIF-1α acted downstream of Caspase14 to mediate tamoxifen resistance. (A and B) Representative western blotting images and densitometric analyses showing the protein levels of HIF-1α, GLUT3, BCRP, Bcl-2, and Bax. Data are presented as mean ± SD (n = 3); *P < 0.05; ***P < 0.001 versus the TAM-R + si-NC group; ^#^P < 0.05; ^##^P < 0.01 versus the TAM-R + si-caspase14 group (two-way ANOVA with post-hoc test for multiple comparisons). (C) Representative images of wound healing assays performed on TAM-R cells transfected with a *HIF-1α*-overexpression plasmid (OE-HIF-1α) or C*ASP14*-specific siRNA (si-caspase14). Scale bar = 200 μm. Data are representative of at least three independent experiments. (D) Cell viability was assessed using the CCK-8 assay in the indicated cell lines and treatment groups after 72 h. Data are presented as the mean ± SD (*n* = 6); ***P < 0.001 versus the TAM-R + si-NC group; ^###^P < 0.001 versus the TAM-R + si-caspase14 group (two-way ANOVA with post-hoc test for multiple comparisons).

HIF-1α overexpression in tamoxifen-resistant MCF7 cells and the knockdown of Caspase14 using siRNA revealed the role of HIF-1α and Caspase-14 in enhancing tamoxifen resistance. Caspase14 knockdown caused a significant decrease in the protein levels of HIF-1α in the MCF7 parental and TAM-R cells (Figure 8A and B). HIF-1α overexpression reversed the decrease in GLUT3 and BCRP protein levels and the BCL2/Bax ratio induced by Caspase14 knockdown (Figure 8 A and B). Additionally, HIF-1α overexpression restored the viability and migratory capacity of the cells, which were inhibited by Caspase14 knockdown (Figure 8C and D). This highlights the potential of targeting the EGR1/Caspase14/HIF-1α pathway as a therapeutic strategy for overcoming drug resistance in breast cancer.

## 4 Discussion

Breast cancer remains a leading cause of cancer-related morbidity and mortality among women globally, characterized by its heterogeneity and complex biology. The development of resistance to endocrine therapies, particularly in the ER + subtypes, poses significant challenges in clinical management. Tamoxifen, a cornerstone treatment for hormone-receptor-positive breast cancer, usually encounters resistance, leading to treatment failure and poor patient outcomes. Understanding the molecular mechanisms underpinning this resistance is crucial to enhancing therapeutic strategies and improving patient prognosis.

We investigated the role of Caspase14 in modulating the EGR1/HIF-1α signaling pathway and its implications for 4-OHT resistance in MCF7 breast cancer cells. By exploring the interplay between Caspase14, EGR1, and HIF-1α, we aimed to elucidate their contributions to the enhanced survival and proliferation of resistant cell populations. The findings highlight the potential of targeting these molecular interactions as a therapeutic strategy to overcome drug resistance, warranting further discussion on the implications of these results for future breast cancer treatment paradigms.

The observed increase in Caspase14 expression within the MCF7 resistant cell lines suggests its pivotal role in modulating resistance mechanisms against 4-OHT. This finding aligns with previous studies indicating that caspases, particularly Caspase14, are implicated in cellular survival and proliferation pathways rather than apoptosis, a notion that contradicts earlier assumptions about caspase function in cancer biology. For example, Denecker et al. emphasized the unique role of Caspase14 in epidermal differentiation rather than in typical apoptotic processes, suggesting that its upregulation enhances cellular adaptability under therapeutic stress conditions [18]. Furthermore, the interaction of Caspase14 with metabolic pathways could provide a survival advantage by promoting glycolytic metabolism, supporting the increased proliferation observed in resistant cells. This metabolic shift, noted in other cancer types, suggests that in addition to traditional pathways, Caspase-14 promotes cell survival through metabolic reprogramming, which is critical in sustaining cellular functions under drug pressure [19]. Our findings extend the emerging paradigm that certain caspases can exert non-apoptotic, pro-tumorigenic functions. Recent studies have highlighted roles for Caspase-3 and -8 in promoting cancer cell survival, migration, and therapy resistance through mechanisms involving metabolic reprogramming and integrin signaling [20]. The upregulation of Caspase-14 in our TAM-R cells suggests it may represent a novel member of this ‘non-canonical’ caspase family contributing to adaptive resistance in breast cancer, warranting comparative functional studies.

In the investigation of the regulatory influence of EGR1 on downstream targets, the downregulation of Caspase14 and associated proteins such as ERα and GLUT3 following EGR1 knockdown underscores its central role in modulating pathways linked to drug resistance. These findings resonate with literature indicating that EGR1 is a transcriptional regulator that coordinates various oncogenic signals, including those that control cell proliferation and migration [21]. The role of EGR1 in upregulating targets involved in drug efflux, such as BCRP, highlights a mechanism through which cancer cells can evade therapeutic effects, further supported by studies illustrating the involvement of EGR1 in enhancing the expression of multidrug resistance genes [22]. This aligns with a recent multi-omics study identifying EGR1 as part of a core transcriptional network activated in in vivo models of endocrine therapy-resistant breast cancer [23]. Our data now provide a direct mechanistic link from this network to the upregulation of specific effector proteins like GLUT3 and BCRP via Caspase-14 and HIF-1α. This indicates a complex interplay between EGR1 and Caspase14, suggesting that interventions aimed at modulating EGR1 synergistically affect Caspase14 levels, influencing therapeutic outcomes in resistant cancer phenotypes.

The HIF-1α upregulation in resistant MCF7 cells correlates with the established knowledge of HIF-1α as a critical factor in promoting tumor aggressiveness and therapeutic resistance through metabolic adaptations. Previous studies have consistently linked HIF-1α overexpression to enhanced glycolytic activity, aligning with our findings of increased glucose uptake and lactate production in resistant cells [24]. Moreover, the ability of HIF-1α to upregulate genes involved in angiogenesis and drug resistance reflects a broader metabolic shift that supports cellular survival in hypoxic conditions, a hallmark of solid tumors [25]. Beyond cell-autonomous metabolic advantages, the HIF-1α-driven lactate overproduction we observed could contribute to an immunosuppressive tumor microenvironment, a key facilitator of therapy resistance that has gained considerable attention recently [26], 27]. This posits the EGR1/Caspase-14/HIF-1α axis as a potential regulator of both intrinsic and microenvironmental resistance mechanisms. This aligns with the notion that HIF-1α may function beyond a survival factor but as a central node in the resistance network, integrating various signaling pathways to promote cell adaptability under therapeutic stress. Such insights imply that targeting HIF-1α could reverse resistance mechanisms, offering a novel therapeutic avenue for overcoming treatment challenges in breast cancer.

The present study demonstrates that the activation of the EGR1/Caspase-14/HIF-1α axis in tamoxifen-resistant cells culminates in the upregulation of key downstream effectors, including GLUT3 and BCRP, accompanied by enhanced glycolytic metabolism. We propose that HIF-1α, as the central transcriptional hub of this axis, coordinately regulates these effectors to drive resistance through synergistic mechanisms. GLUT3 facilitates increased glucose uptake, fueling the elevated glycolytic flux and lactate production, thereby providing the necessary bioenergetic and biosynthetic support for proliferating drug-resistant cells [28], 29].Concurrently, BCRP (ABCG2), a well-characterized multidrug efflux pump, is postulated to actively export 4-OHT, directly reducing its intracellular concentration and conferring pharmacologic resistance [30], 31]. The critical evidence supporting their functional indispensability stems from our rescue experiment (Figure 8), wherein HIF-1α overexpression reversed the suppression of GLUT3/BCRP and the associated resistant phenotypes induced by Caspase14 knockdown.

We acknowledge that a direct causal demonstration – such as showing that enforced expression of GLUT3 or BCRP alone is sufficient to induce tamoxifen resistance in parental cells – remains a limitation of the current study. Future investigations employing such gain-of-function approaches, as well as targeted inhibition of GLUT3 or BCRP in resistant cells, will be essential to definitively establish their necessity and sufficiency within this network. Nonetheless, our findings nominate the HIF-1α/GLUT3/BCRP module as a critical downstream executive arm of the novel EGR1/Caspase-14-driven pathway, offering tangible targets for therapeutic intervention.

The significant enhancement in migration capacity observed in resistant MCF7 cells could be intricately linked to the regulatory interplay between Caspase14 and HIF-1α. This observation corroborates existing literature that highlights the role of Caspase14 in modulating cellular migration and invasion through its influence on cytoskeletal dynamics and extracellular matrix interactions [32]. The interplay between metabolic shifts induced by HIF-1α and the migratory cues regulated by Caspase14 could create a permissive microenvironment for tumor progression. Conversely, strategies that inhibit Caspase14 expression impede migration in other cancer types, suggesting that its role in migration may be context-dependent and influenced by surrounding cellular signals [33]. BCRP and GLUT3 function indirectly yet are essential contributors to the migratory phenotype. By mediating drug efflux and fueling glycolysis, they sustain cell survival and metabolic adaptation, thereby maintaining a viable cell population competent for migration [34], 35]. More directly, the EGR1/Caspase-14/HIF-1α axis activity promotes an anti-apoptotic shift (elevated Bcl-2/Bax ratio), which enhances resistance to detachment-induced anoikis, a critical permissive factor for successful cell migration [36]. Ultimately, the most direct effectors of the migratory phenotype are the HIF-1α-driven transcriptional programs, which upregulate genes involved in cytoskeletal remodeling, extracellular matrix degradation, and epithelial-mesenchymal transition (EMT), thereby actively propelling cell motility [37]. This underscores the necessity for further exploration into the mechanistic pathways linking these proteins to enhance the understanding of their contributions to tumor metastasis and therapeutic resistance.

The metabolic alterations observed in resistant MCF7 cells, characterized by increased glucose consumption and lactate production, align with the concepts of metabolic reprogramming in cancer. This phenomenon is well-documented in the literature, where enhanced glycolysis is usually linked to survival under therapeutic duress, further supported by studies demonstrating the relevance of metabolic adaptations in various cancer types [38]. Notably, the interaction between Caspase14 and metabolic pathways may indicate a dual role of Caspase14 in promoting survival and facilitating metabolic shifts that sustain drug resistance. Targeting metabolic pathways reportedly sensitizes resistant cancer cells to therapies, suggesting that Caspase14 represents a therapeutic target that could disrupt this metabolic reprogramming, enhancing treatment efficacy [39]. These findings may aid further investigations aimed at understanding the metabolic dynamics in breast cancer resistance.

Beyond delineating the mechanistic axis, an important translational consideration raised by our findings is the relative merit of EGR1 versus Caspase-14 as a therapeutic target for overcoming tamoxifen resistance. While our data unequivocally establish EGR1 as a critical transcriptional mediator within this pathway – its knockdown effectively suppresses Caspase-14, GLUT3, BCRP, and the resistant phenotype – targeting EGR1 directly presents significant challenges. EGR1 is an immediate-early response transcription factor with pleiotropic roles in diverse fundamental processes, including cellular proliferation, differentiation, apoptosis, and stress adaptation across many tissue types [40]. Systemic inhibition of such a multifunctional protein could therefore lead to substantial on-target, off-tumor toxicity, limiting its therapeutic window. In contrast, Caspase-14 exhibits a more restricted expression profile, predominantly associated with epithelial differentiation and having fewer documented roles in broad physiological processes compared to EGR1 [41]. This narrower functional scope suggests that targeting Caspase-14 may offer a more favorable safety profile with reduced risk of unintended systemic effects. Furthermore, within the context of our proposed model where Caspase-14 activation sits upstream of EGR1 and HIF-1α upregulation, pharmacological inhibition of Caspase-14 could potentially disrupt the entire pathogenic loop at its initiation point, thereby simultaneously attenuating the activity of both EGR1 and HIF-1α. This upstream intervention might be more efficacious than targeting individual downstream components. Therefore, while both nodes are mechanistically integral, Caspase-14 emerges as a potentially more viable and specific direct therapeutic target. Future studies employing selective Caspase-14 inhibitors, alongside strategies aimed at disrupting the EGR1/Caspase-14 interaction or feedback loop, will be crucial to validate this hypothesis and translate these findings into novel therapeutic strategies for breast cancer.

**Figure 9:**
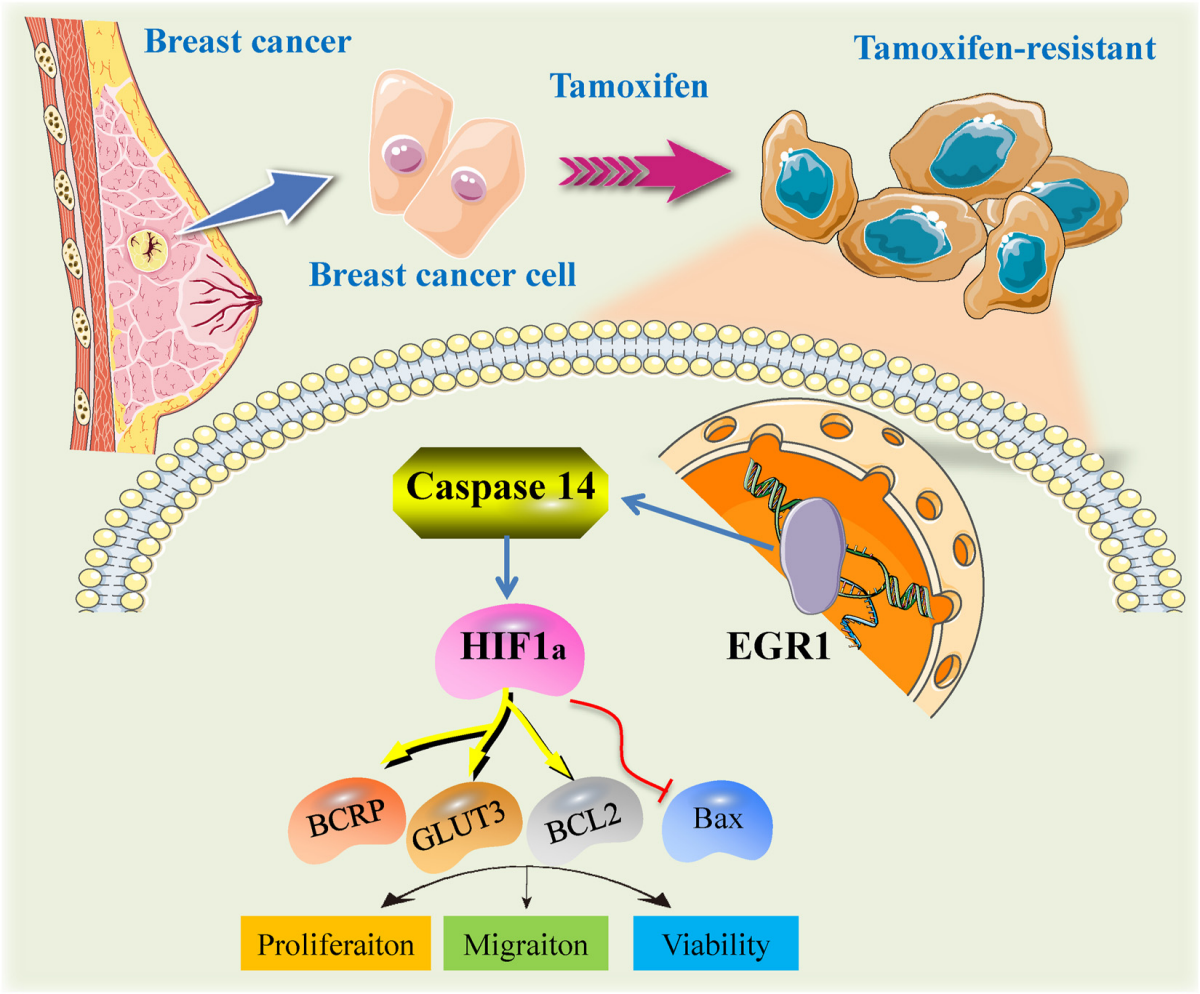
Schematic summary of key molecular components and phenotypes associated with the EGR1/Caspase-14/HIF-1α axis in tamoxifen-resistant MCF-7 breast cancer cells. This illustration conceptualizes the core findings of this study. The development of tamoxifen resistance in breast cancer cells involves the upregulation of Caspase-14, which is linked to the activation of transcription factors EGR1 and HIF-1α. Downstream effectors of this axis include the drug efflux pump BCRP (ABCG2), the glucose transporter GLUT3, and altered expression of apoptosis regulators (BCL-2 and Bax). Collectively, these molecular changes contribute to the enhanced cellular phenotypes observed in resistant cells, including increased proliferation, migration, and viability.

This study elucidates the regulatory role of Caspase14 in the EGR1/HIF-1α signaling pathways and their collective impact on the drug resistance of MCF7 breast cancer cells to 4-OHT. The identification of Caspase14 as a potential therapeutic target is innovative, suggesting new avenues for overcoming treatment resistance. A schematic summary of this proposed mechanism is presented in Figure 9. Future studies should focus on exploring the clinical applicability of these findings, including the development of targeted therapies that effectively inhibit Caspase14 or disrupt the EGR1/HIF-1α axis, improving treatment outcomes for patients with resistant breast cancer.
